# Human Herpesvirus-8 Infection Associated with Kaposi Sarcoma, Multicentric Castleman's Disease, and Plasmablastic Microlymphoma in a Man with AIDS: A Case Report with Review of Pathophysiologic Processes

**DOI:** 10.4061/2011/647518

**Published:** 2010-12-30

**Authors:** Christian Eaton, Russell Dorer, David M. Aboulafia

**Affiliations:** ^1^Department of Medicine, Virginia Mason Medical Center, Seattle, WA 98101, USA; ^2^Department of Pathology, Virginia Mason Medical Center, Seattle, WA 98101, USA; ^3^Division of Hematology and Oncology, Virginia Mason Clinic, Seattle, WA 98101, USA; ^4^Division of Hematology, University of WA, Seattle, WA 98101, USA

## Abstract

Kaposi sarcoma (KS), multicentric Castleman's disease (MCD), and plasmablastic microlymphoma, are all linked to human herpesvirus-8 (HHV-8) infection and HIV-induced immunodeficiency. Herein, we describe the case of a Kenyan man diagnosed with HIV in 2000. He deferred highly active antiretroviral therapy (HAART) and remained in good health until his CD4+ count declined in 2006. He was hospitalized with bacterial pneumonia in 2008, after which he agreed to take HAART but did so sporadically. In 2010, he was hospitalized with fever, lymphadenopathy, pancytopenia, and an elevated HHV-8 viral load. A lymph node biopsy showed findings consistent with KS, MCD, and plasmablastic microlymphoma. Eight months after starting liposomal doxorubicin, Rituximab, and a new HAART regimen, he has improved clinically, and his HIV and HHV-8 viral loads are suppressed. These three conditions, found in the same lymph node, underscore the inflammatory and malignant potential of HHV-8, particularly in the milieu of HIV-induced immunodeficiency.

## 1. Introduction

In the current era of highly active antiretroviral therapy (HAART), people infected with HIV are not only less likely to develop AIDS-defining infections, they are also less likely to be diagnosed with an AIDS-defining neoplasm (i.e., Kaposi Sarcoma (KS) or non-Hodgkins lymphoma (NHL)) [1–3]. As these individuals live to their sixth decade of life and beyond, cancers associated with lifestyle choices and aging (i.e., lung, liver, and anal carcinomas) are increasingly important barriers to survival [4–6]. However, AIDS-defining malignancies remain a significant cause of morbidity and mortality for those individuals who are not on HAART because they are unaware of their HIV serostatus, they do not have access to HAART, or they are poorly adherent with prescribed therapies [7].

KS is etiologically linked to human herpesvirus-8 (HHV-8), also known as KS-associated herpesvirus (KSHV) [8]. The HHV-8 genome contains numerous genes that code for proteins with recognizable homology to human proteins, including an interleukin-6 (IL-6) homologue [9]. IL-6 has multiple systemic effects including the support of hematopoiesis and stimulation of B lymphocyte and plasma cell growth. When expressed in physiologic excess, IL-6 may contribute to dysregulation of immune responses [10].

IL-6 is also a major mediator of the systemic symptoms associated with Castleman's disease (CD) [11, 12]. CD is a heterogeneous group of lymphoproliferative disorders of unknown etiology. Unlike unicentric CD, multicentric CD (MCD) is strongly associated with immunosuppression and HHV-8 infection [13]. Patients with MCD frequently present with generalized lymphadenopathy, hepatosplenomegaly, fever, and night sweats. These debilitating systemic symptoms are in part due to the proinflammatory effects of IL-6 [14]. IL-6 also downregulates albumin production by the liver, leading to hypoalbuminemia, which may cause anasarca via decrease in oncotic pressure.

When comparing HIV-infected patients without MCD, those with MCD have a 15-fold increased risk of NHL, with the most common subtype of NHL being HHV-8-positive plasmablastic lymphoma. Among 60 patients with HIV and MCD, 14 were diagnosed with NHL of whom 6 (43%) had the plasmablastic variant [15]. In some cases, the precursor to HHV8+ plasmablastic lymphoma are the sheets of plasmablastic cells, termed microlymphomas, found in the lymph nodes of MCD patients [15, 16]. These microlymphomas typically express IgM with lambda light chain restriction, but are polyclonal whereas true HHV8+ plasmablastic lymphoma is monoclonal and follows an aggressive clinical course, responding poorly to multiagent chemotherapy [16].

Herein, we describe the case of an HIV-infected man with KS, MCD, and microlymphoma, all three of which were identified within a single resected lymph node. We also review the relevant literature with a focus on the common etiology of these various diseases.

## 2. Case Report

In 2000, a 42-year-old Kenyan man, who had sex with other men, was diagnosed with HIV infection and neurosyphilis while he was being evaluated for chronic sinusitis and headache. His initial CD4+ count was 595 cells/*μ*l, and his HIV viral load was 5,490 copies/ml. Neurosyphilis was successfully treated, but the patient opted to defer HAART. In 2003, he had unilateral and painless right thigh enlargement and underwent surgical excision of a low-grade leiomyosarcoma. No adjuvant treatment was recommended, and he remained in good health for an additional two years, during which time his CD4+ count and his HIV viral load remained >550 cells/*μ*l and <5,500 copies/ml, respectively.

**Figure 1:**
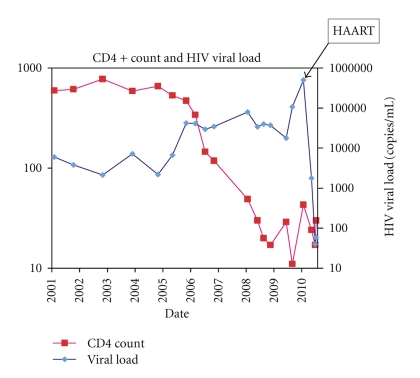
Graph of CD4+ cell counts and HIV viral loads from 2001 to 2010 plotted on a logarithmic scale showing the increase in the viral load heralding the fall in CD4+ count, as well as showing the drop in the viral load after the patient became compliant with HAART.

In 2006, the patient had a precipitous and persistent fall in CD4+ count to <120 cells/*μ*l and his HIV viral load increased to 35,000 copies/ml. However, he declined to begin HAART for fear of untoward medication-associated side effects. Over the next two years he lost 15 pounds, had waxing and waning adenopathy, and was hospitalized on one occasion with bacterial pneumonia. By June, 2008 he had greater than 15 discrete KS lesions on his legs, his CD4+ count was 30 cells/*μ*l and his HIV viral load was 34,000 copies/ml (Figure 1).

In 2009, he was hospitalized for treatment of methacillin-susceptible staphylococcus aureus pneumonia and bacteremia. He subsequently agreed to begin HAART, but was poorly adherent with treatment, stopping and restarting his HIV medications on a number of occasions.

In April 2010, he was hospitalized with intermittent fevers, night sweats, mild pancytopenia, hypoalbuminemia, and anasarca. A computerized tomogram (CT) of chest, abdomen, and pelvis revealed diffuse but modest adenopathy. He was ultimately discharged to home when assorted cultures and special studies returned negative for an acute infection. He was strongly encouraged to take HAART, now consisting of darunavir, etravirine, raltegravir, and ritonavir.

**Figure 2:**
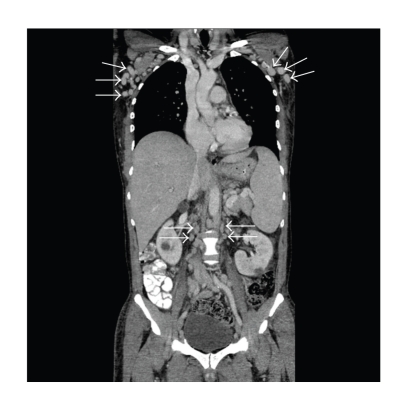
Coronal computerized tomograms of chest, abdomen, and pelvis with arrows highlighting foci of axillary and retroperitoneal adenopathy.

**Figure 3:**
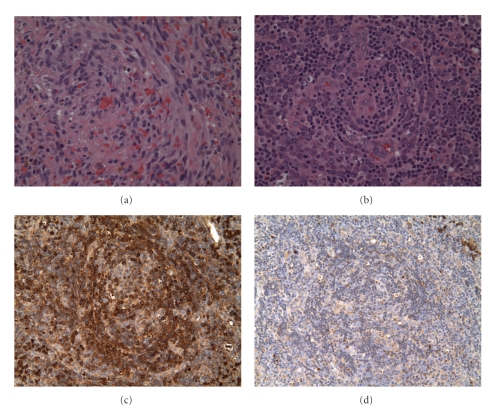
(a) H&E at 200x, illustrating typical Kaposi sarcoma morphology, (b) 100x of residual follicle with plasma cells, plasmablasts and mixed inflammation, (c) lambda light chain immunohistochemistry (IHC) at 100x, D) kappa light chain IHC at 100x.

One month later he was readmitted with recrudescent fevers, palpable axillary and groin adenopathy, worsening anasarca, and more numerous KS lesions on his neck, trunk, and legs. Laboratory studies were notable for the following, WBC 2400/mm^3^; hematocrit 24%, platelet count 48,000 cells/mm^3^, alkaline phosphatase 409 units/L (normal <150 U/L), lactate dehydrogenase 636 units/L (normal <243 U/L), and serum albumin 2.9 g/dL, and a serum protein electrophoresis revealed elevated globulins but no monoclonal gammopathy. His CD4+ count was 24 cells/*μ*l, HIV viral load 1,792 copies/ml, and HHV-8 viral load was 10,000,000 copies/ml. A repeat CT scan showed moderate progression of adenopathy which was most prominent in the axilla and retroperitoneum (Figure 2). An axillary lymph node was biopsied and showed changes in keeping with MCD. In addition, numerous B cell follicles contained broad mantle zones with numerous larger plasmablastic cells that formed microscopic clusters and sheets within the germinal centers and in the interfollicular areas. These plasmablastic cells expressed HHV-8 and lambda light chain consistent with so-called microlymphoma, which was supported by additional studies. A focus of HHV-8 positive KS spindle cells was also identified (Figures 3(a)–3(d)). 

The patient, now age 52, began treatment consisting of liposomal doxorubicin (20 mg/m^2^ every 14 days) in conjunction with Rituximab (375 mg/m^2^ weekly times four) and concurrent HAART consisting of the same regimen listed above. More aggressive treatment with multiagent chemotherapy was not given since the patient had only premalignant plasmablastic microlymphoma, and not frank HHV8+ plasmablastic lymphoma, and MCD and KS have been shown to respond to this more conservative treatment.

At eight-months followup, anasarca had resolved and he had regained lost weight. Cutaneous KS was less prominent, and a CT scan showed diminished adenopathy. Laboratory studies are now notable for a gradual improvement in complete blood count, hepatic transaminases, and alkaline phosphatase. His serum albumin has returned to normal, his HIV viral load is nondetectable; although, his CD4+ count remains <50 cells/*μ*L, and his HHV-8 viral load has decreased by greater than two orders of magnitude.

## 3. Discussion

The three separate conditions of KS, MCD, and plasmablastic microlymphoma, all identified within a single lymph node biopsy in a patient with AIDS, underscore the diverse malignant and inflammatory potential of HHV-8 infection, especially in the milieu of decreased cellular immunity due to HIV infection.

HHV-8 is a member of the gammaherpesvirus family and shows 40% sequence homology with the oncogenic Epstein-Barr virus [17]. Like other herpesviruses, HHV-8 is adept at evading the immune system through its ability to use viral immunomodulators that interfere with the host immune response, many of which are homologues of human genes [18]. One way HHV-8 evades the immune system is through the skewing of the host immune response from Th1 to Th2, in part accomplished by the action of viral-IL-6 (vIL-6). This homologue of human IL-6 acts through gp130 to promote Th2-cell development and responsiveness, while also inhibiting Th1-cell responses [19]. HHV-8 also encodes proteins that interact with the host immune system to inhibit complement and down regulate the adaptive immune response.

The oncogenic potential of HHV-8 is, in part, due to its ability to cause chromosomal instability, to alter gene expression, to increase telomerase activity, and, promote cell invasiveness, proliferation, and survival [20]. The latency-associated nuclear antigen (LANA) inhibits p53-induced apoptosis while also inactivating the retinoblastoma gene, which would otherwise inhibit progression through the G1/S cell cycle checkpoint [21, 22]. vIL-6 can activate multiple cellular pathways, including JAK/STAT, which in turn leads to vascular endothelial growth factor expression and signaling [23]. When vIL-6-expressing fibroblasts are injected into mice, highly vascular tumor formation, hematopoiesis, and plasmacytosis take place [24]. Other viral genes code for proteins that are also implicated in oncogenesis: viral interferon regulatory factor-1 (v-IRF-1) suppresses both type I and type II interferon responses; K13 (aka vFLIP) inhibits Fas-mediated apoptosis; viral g-protein-coupled receptor (vGPCR), a homologue of the IL-8 receptor, produces tumors when vGPCR-expressing fibroblasts are injected into nude mice [25–28].

Since Chang and colleagues first identified HHV-8 in KS tissue from HIV seropositive patients, our understanding of the pathophysiology of various HHV-8-associated diseases has grown substantially [29]. HHV-8 is now etiologically linked to KS in HIV-infected patients, as well as KS in immunocompetent patients (classic KS) [30]. More recently, HHV-8 has been associated with MCD, HHV8+ plasmablastic lymphoma, primary effusion lymphoma, and germinotrophic lymphoproliferative disorders [31–33].

HIV-seropositive individuals with MCD have a significantly greater risk of NHL than their HIV-negative counterparts. In MCD, HHV-8 is specifically associated with monotypic (IgM *λ*) but polyclonal HHV-8+ plasmablasts which occur as isolated clusters of cells in the mantle zone of B-cell follicles, called microlymphomas [34, 35]. The expansion of these plasmablastic microlymphomas from MCD lesions to aggressive NHL is probably triggered by a second oncogenic event. This implies that MCD, microlymphoma and HHV8+ plasmablastic lymphoma represent three stages of a single disease in the backdrop of HIV-induced immunodeficiency.

The specific NHL implicated in this process, HHV-8+ plasmablastic lymphoma, is a rare, aggressive B-cell lymphoma with a poor prognosis [36]. In an abstract regarding 131 patients with AIDS-associated plasmablastic lymphoma, death occurred in 59% of patients with a median survival of only 14 months from diagnosis [37]. However, only 16% of those 131 cases were HHV-8+, and these were later excluded from the final, peer-reviewed publication [38], highlighting the fact that the HHV-8+ form of plasmablastic lymphoma is distinct from the more common plasmablastic lymphoma, which is more frequently associated with Epstein-Barr virus and often presents as skin or oropharyngeal nodules. The distinction between these different forms of plasmablastic lymphoma, and any possible differences in clinical course and response to treatment, is an area where further research is needed.

Coexistence of MCD and KS in the same tissue is a common phenomenon. Among 24 lymph nodes, 15 (63%) showed evidence of coexisting KS [39]. The association may be due to lytic HHV-8 infection of B-lymphoid cells exposing susceptible endothelial cells at vulnerable sites. Disregulation of IL-6 in HHV-8-infected cells is not only a major trigger for disease in the angiofollicular hyperplasia of MCD, and KS, it is also a major trigger for disease progression in HHV-8_plasmablastic and primary effusion lymphomas. 

While the overall median survival of patients with AIDS and coexistent MCD and NHL is generally just a few months, there are exceptions [40, 41]. Horster and colleagues describe the case of a patient with AIDS, MCD and plasmablastic leukemia who was treated with multiagent chemotherapy, splenectomy, and maintenance thalidomide and who was still alive at a 28-month followup [42]. Another patient with AIDS and MCD had a plasmablastic lymphoma of the spermatic cord. He too was treated with multiagent chemotherapy, and despite a subsequent relapse of MCD he was alive at an 11-month followup [43]. Given recent successes associated with treating patients with HIV-associated MCD with antivirals, and the better outcome associated with treating AIDS-related lymphoma patients with modern supportive care in conjunction with HAART, rituximab, and when needed, chemotherapy, we are hopeful that the prospects of HIV-infected patients with HHV-8-associated plasmablastic lymphoma, KS, and MCD will also improve [44, 45].
